# Reproductive justice and the Figure of the Child: The multiple harms of forced sterilization and abortion in Peru

**DOI:** 10.1002/fea2.12124

**Published:** 2023-08-30

**Authors:** Julieta Chaparro‐Buitrago, Cordelia Freeman

**Affiliations:** ^1^ Department of Sociology Reproductive Sociology Research Group University of Cambridge Cambridge UK; ^2^ Department of Geography University of Exeter Exeter UK

**Keywords:** figure of the child, abortion, forced sterilization, Peru, reproductive justice

## Abstract

We argue that discourses around forced sterilization and abortion restrictions too often focus on motherhood and fertility and ignore the multiple other harms they bring. To do this, we travel with the North American framework of reproductive justice (RJ) to think through experiences of (non)reproduction in Peru and consider its analytic possibilities. In this intervention, we wish to focus on the commonality between RJ's three tenets: the figure of the child and its analytic force. We argue that while the aim of the RJ framework is not to reify fetuses and children at the expense of adults nor to reinforce a pronatalist position, the fact that the tenets are formulated around children means that, when mobilized for political or analytical purposes, they can reinforce repronormative mandates. We use the examples of forced sterilization and abortion in Peru to consider the issues this figuration of the child brings into being and the landscape of meaning it produces.

## INTRODUCTION

Peru is a country marred by reproductive injustice. Across all areas of fertility and reproduction—access to contraception, sex education, access to abortion, maternal health, obstetric violence, and birthing experiences—there are deep, structural inequities in experiences and outcomes. These inequities fall most heavily on poor, racialized, Indigenous, and rural women. The confluence of colonialism, heteropatriarchy, classism, and combining traditional Catholicism with neoconservative evangelicalism has strengthened pro‐maternal ideology with violent effects on women.

Forced sterilization and abortion restrictions are both forms of reproductive injustice that disproportionately affect the lives of poor, rural, and Indigenous women in Peru. For the last three decades, reproductive politics have been at the forefront of public debates and controversies in Peru (Cáceres et al., 2008), primarily around feminist demands for abortion legalization, access to contraceptives, and gender equality. The historic feminist demand for the expansion of contraceptives was, in a tragic turn of events, crystalized during the second half of the 1990s at the time when a mass sterilization campaign unfolded nationwide under the auspices of the Reproductive Health and Family Planning Program 1996–2000 (RHFPP). During the same time, initiatives to decriminalize abortion were truncated after a staunch attack by the Catholic Church and conservative politicians on attempts to reform the 1924 Criminal Code that banned abortion except for therapeutic reasons (Cáceres et al., 2008).

We argue that discourses around forced sterilization and abortion restrictions too often focus on motherhood and fertility and ignore the multiple other harms they bring. To do this, we travel with the North American framework of reproductive justice (RJ) to think through experiences of (non)reproduction in Peru and consider its analytic possibilities. Reproductive justice was born from Black feminists’ theorization and reflection on reproductive injustices. It proposes three key tenets: the right to have children, the right not to have children, and the right to parent children in safe and healthy environments (Ross & Solinger, 2017). In this intervention, we focus on the commonality between those three tenets: the figure of the child and its analytic force. We argue that while the aim of the RJ framework is not to reify fetuses and children at the expense of adults nor to reinforce a pronatalist position, the fact that the tenets are formulated around children means that, when mobilized for political or analytical purposes, they can reinforce repronormative mandates. We use the examples of forced sterilization and abortion in Peru to consider the issues this figuration of the child brings into being and the landscape of meaning it produces.

Questioning the figure of the child in reproductive justice is not to disavow the history of reproductive dispossession that has stripped the possibility for racialized women to have children and parent them. We want to avoid the trap of disregarding the centrality of race in intersectional analysis, which can erase the centrality of Black women's experience in conceptualizing reproductive justice. However, we still need to question how the *figure of the child* (not of children and parents) shapes our understanding of reproductive abuse and certain framings about abortion. This commentary builds on Castañeda's (2002) work on child figuration. As she notes, although this figuration “bears on actual children and their experiences of the world, … [it] is not about that relation” (3). Instead, this figuration allows the possibility of “making wider cultural claims” (3) about the world, bodies, and practices and constructing “facts” about human nature. That said, we suggest that the scholarship on reproductive justice would benefit from paying attention to the lives of children and the conditions that sustain their lives.

The goal of this piece, then, is not to ignore histories of forced removal of children or forced sterilization but to examine the figure of the child as a symbolic place where questions of motherhood and womanhood are reified in abortion and forced sterilization politics. Building on our research in Peru, we explore how this figuration confines our understanding of forced sterilization to fertility loss, preventing us from grasping the multiple and interconnected harms that unfold in people's lives. Likewise, we examine how abortion discourse can fail to consider the harms of abortion restrictions beyond existing or future children and instead focus disproportionately on ideals of “good motherhood.”

## FORCED STERILIZATION AND THE PHANTASMAGORIC CHILD

Reproductive justice thinkers have been critical of reproductive rights as it “advocate[s] almost exclusively for the legal right to abortion, further distancing its agenda from the interests of women who have been the targets of sterilization abuse because of the devaluation of their right to bear children” (Roberts, 2015, 79). This proposition is fundamental to reproductive justice as histories of forced sterilization are central to the formulation of its three tenets. Alongside welfare reform and the foster system, coercive sterilizations of Black, Native American, and Latinx women constitute “official reproductive abuse of people of color and their communities” (Ross & Solinger, 2017, 14). Despite its centrality, reproductive violence and the harms it causes remain undertheorized in reproductive justice scholarship.

Recent work in feminist scholarship on transitional justice can help us in this regard. Ciara Laverty and Dieneke de Vos (2021) question the folding of reproductive violence under wartime rape. To disentangle them, the authors describe the motive behind reproductive violence as “a violation of reproductive autonomy … directed at people because of their reproductive capacity” (4). It is clear that the driver behind reproductive violence is controlling people's fertility; however, to assume that harms it inflicts on people's lives are limited to it is problematic. Similarly, other forms of violence, like rape, may cause reproductive harm, such as forced pregnancies. In their words, “what it means for a violent act to be [reproductive] is to some extent not fixed” (Laverty & de Vos, 2021, 7).

Their critique offers insights that can help reproductive justice's theorization of reproductive violence and the harms that unfold in people's lives. Reproductive justice's broader agenda of social justice provides an entry point to understand better the harms that women have to negotiate in the aftermath of violence, revealing the entanglement of reproduction with other dimensions of women's lived experience such as labor, sexuality, understanding of the body, and community. The goal, then, is to enrich reproductive justice with categories and language to consider such harms instead of taking them for granted.

The Peruvian case of forced sterilization is ideal for illustrating this argument. In 1995, President Fujimori launched the Reproductive Health and Family Planning Program 1996–2000, which was allegedly going to make various birth control methods (except abortion) available for all Peruvian women to guarantee their bodily autonomy and empowerment. This promise turned out to be the exact opposite. RHFPP became the platform for forcibly sterilizing peasant, Indigenous, and low‐income women nationwide. The Peruvian case is part of a global history of eugenics and population control that subjected the bodies of certain groups of women, often racialized and impoverished, to the imperatives of racial hygiene and the “economization of life” (Murphy, 2017).

What are the figurations through which reproductive abuse and harm are made intelligible? The figure of the child, even if in ghostly form, is one of them. Contrary to the visual imagery of the fetus in antiabortion campaigns, the ghostly child is the nonconceived one that haunts the potential living mother. Similarly to the abortion debate, forced sterilization is often perceived as truncating women's trajectories of motherhood, considered desirable, if not mandatory. Yet sterilization abuse is exercised on those bodies whose reproduction is devalued. The focus on fertility loss and the impossibility of biological reproduction obscures the fact that many survivors are already parents to other children or that some women desire to curtail their fertility or feel ambivalent about mothering. None of these facts, however, legitimize sterilization abuse.

The phantasmagoric child sways our understanding of reproductive abuse, obscuring other harms that do not neatly fall within fertility‐centric narratives. Research with survivors of the Peruvian sterilization campaign shows that women use a grammar of reproductive abuse in which fertility loss is one of a variety of harms, including the loss of physical strength, the idea that the sterilization alters their sexual behavior, compounding impoverishment, hunger, and conflicts within their families and communities (Chaparro‐Buitrago, 2022). Women's narratives weave infertility together with a whole constellation of injuries that exceed the fixed boundaries set up by reproductive rights, understood almost exclusively as the ability to control one's fertility (Chaparro‐Buitrago, 2022). This is partly the result of reproductive rights’ normative prescription around responsible parenthood built on the idea of proper fertility regulation. Good parents responsibly decide on the number of children and the spacing of births to guarantee a good life for them. This articulation raises questions about who is perceived as a responsible parent, considering historical depictions of Indigenous/peasant women as lacking intelligence, reason, or common sense (de la Cadena, 2000).

This argument has broader implications for possible reparations for survivors. The question is, what harm(s) should the government repair? Historian Donna Drucker's discussion on legal redress is exemplary. Fujimori, she reminds us, is currently in detention for corruption and human rights violation charges—although it is important to clarify not for the forced sterilization case—“but his punishment does not restore victim's fertility” (Drucker, 2020, 63). Her statement centers on infertility as the main harm, erasing all the others we just mentioned that constitute major issues survivors have had to negotiate and live within the afterlife of sterilization abuse. We argue that RJ scholarship and activism need to be attentive to framings of reproductive violence that reduce it to infertility to capture a broader range of harms and injustices, including, but not limited to, the inability to bear children. In fact, women's narratives echo reproductive justice's premise that reproductive rights are not enough. A broad social justice agenda is necessary, including economic and racial justice, for families, individuals, and communities to satisfy their reproductive preferences.

## ABORTION, GOOD MOTHERHOOD, AND THE FETISHIZATION OF THE FETUS

Abortion in Peru is highly restricted and criminalized. Someone who accesses an abortion can be sentenced to up to 2 years in prison, and anyone who performs one can be sentenced to up to 5 years (Cámara‐Reyes et al., 2018). Technically, “therapeutic abortions” when there is a severe risk to the pregnant person's health are legally available, but in reality, they are highly restricted and inaccessible. These restrictions make the number of abortions in Peru challenging to count, but we know they are highly common, with estimates of 350,000 per year (Cáceres et al., 2008). The Peruvian state's refusal to acknowledge abortion means that the vast majority of these abortions occur clandestinely. It is important to note that this does not mean all are unsafe, as feminist activism around the abortion pill misoprostol and the extensive networks of providers that offer surgical abortions have seen a reduction in maternal mortality related to unsafe abortion (Duffy et al., 2023).

“Family planning” and fertility control are dominant in Peru through “compulsory motherhood” and the discourse that it is always a “blessing” (Seperak Viera et al., 2019). Gender relations in Peru are heavily determined by Catholicism and the idea of *marianismo*, the feminine ideal that emphasizes the importance of motherhood, submissiveness, and martyrdom for one's family (Boesten, 2010). Being a good Peruvian woman is merged with being a good mother, so reproductive autonomy is quashed in favor of pronatalism and the reproduction of future Peruvian citizens, particularly white ones (Boesten, 2007). Poor access to contraception and sex education, along with high levels of sexual violence but no recourse to end an unwanted pregnancy, enforce compulsory motherhood. By enshrining the fetus with legal personhood, the autonomy of pregnant people is made nonexistent, and they are compelled to continue with a pregnancy even if they have no desire to, if the fetus is inviable, or if the pregnant person is a child. The only righteous aborter, then, is the one who would otherwise die; all others must be the self‐sacrificing maternal figure. Peru is not alone in this. “Good motherhood” is frequently espoused as an argument for abortion access with reasons such as “it's not the right time for me yet,” the aspiration to parent when more financially stable, or the desire to focus parenting energies on already‐existing children. All reasons for wanting an abortion are valid, and any distinction between “good” and “bad” abortions must be challenged, but there is more stigma around sharing non‐child‐focused reasons such as “I just didn't want to be pregnant.”

We identify two ways abortion discourse can be reframed to move beyond “good motherhood.” The first is by expanding the language and framing to understand abortion. In her book *Happy Abortions* (2017), Erica Millar theorizes a radically different conceptualization by celebrating abortion. She critiques the obsession with the maternal subject in abortion politics and argues that the limited range of emotions available for people to describe their abortions (grief, guilt, shame) is fundamentally antiabortion. The schema of fetal motherhood must be resisted to center the people who have abortions and the full spectrum of emotions that they experience. In Peru, abortion activists are embodying this resistance and shifting abortion discourse beyond the figure of the child. They challenge the notion of good motherhood by celebrating abortion and resisting state governance of reproductive autonomy. For instance, *acompañante* networks are groups of varying size and formality that support people, emotionally, physically, and practically, to have abortions in restrictive contexts in Latin America. Through this accompaniment, abortion can be reimagined as a collective endeavor that rejects the state's compulsory motherhood and offers love, support, and care (Duffy et al., 2023). These activists refuse the justification of abortion under socially acceptable norms and the narrow limitations that the state imposes and instead celebrate abortion on the terms people desire.

Second, to push abortion discourse beyond the fetishization of the fetus, it is important to consider the harms of abortion restrictions beyond existing or future children. Aideen O'Shaughnessy (2022, 76) developed the term “abortion work” to refer to the deeply inequitable “emotional and material labor which is unequally imposed on women and gestational subjects in Ireland, as they negotiate and plan for the possibility of needing to acquire an (il)legal abortion, either inside or outside the state.” In Peru, the restrictions on abortion result in this anticipatory abortion work that might include keeping savings for an abortion, knowing how to access an illegal abortion, and expending emotional energy on the concerns of needing an abortion in a state that denies access. O'Shaughnessy's work allows us to consider the broad impact that abortion criminalization has on people's lives in material and emotional ways that go far beyond the figure of the child.

Reproductive justice scholarship and activism are highly effective at calling attention to the entangled nature of people's reproductive lives and have attempted to challenge narrow expectations of “good motherhood” that have been built around class and race privileges (Ross & Solinger, 2017). Nevertheless, the right not to have a child, and the right to have an abortion specifically, must not be exclusively tied to being a good parent in the present or the future. The culture of compulsory motherhood in Peru fetishizes the fetus as a future citizen and obligates the continuation of all pregnancies. The pervasive figure of the child inadvertently pulls attention away from the person having the abortion, so we therefore call for the celebration of abortion in itself, the centering of the people having abortions, and the recognition of the harms of abortion restrictions that go beyond the figure of the child.

## CONCLUSION

We have traveled with reproductive justice to consider abortion and forced sterilization politics in Peru. The Peruvian context has brought to the fore the relevance of reproductive justice for thinking about Latin American realities. In turn, it provides important insights for understanding that achieving reproductive justice in a deeply unjust context such as Peru requires challenging dominant narratives of good motherhood and the unequal valuation of people by their fertility. Reproductive justice is crucial in a place where the state has inflicted harm and control on the reproductive lives of people across the country in contradictory ways, both by enforcing motherhood or denying the right to parenting, particularly for marginalized communities.

The figure of the child, however, raises some questions for reproductive justice scholarship. As Ross and Solinger (2017, 306) suggest, “reproductive justice thrives in the borderlands of ambiguity, and its incompleteness offers amazing flexibility and adaptability to allow multiple interpretations that invite elaboration and clarification.” This is a benefit as well as a challenge. In this piece, we discussed repronormative elements that undergird how abortion and sterilization politics are constructed, either as a postponement or impeding of motherhood, and reproductive justice was not helpful in calling them into question. Even though it makes a powerful argument about social justice as a condition for reproductive freedom, RJ is less productive when addressing these repronormative scrips, as its three main tenets revolve closely around children. We also argue that child figuration can limit the boundaries of imagining true reproductive freedom. When we limit reproduction to biological reproduction, we also limit the epistemological possibilities reproduction can offer and the issues we pay attention to. Reproductive justice has built a fertile ground for expanding what reproduction is and what is needed to make it possible. Environmental, racial, economic, migrant, and social justice are fundamental to sustaining life and kin‐making. Hence, this commentary highlights the normative assumptions around reproduction and motherhood, concentrated around the figure of the child that can restrict an expansive agenda for reproductive justice.

## CONFLICT OF INTEREST STATEMENT

The authors report no competing interests to declare.

## References

[fea212124-bib-0001] Boesten, J. 2007. “Free Choice or Poverty Alleviation? Population Politics in Peru under Alberto Fujimori.” European Review of Latin American and Caribbean Studies 82: 3–20.

[fea212124-bib-0002] Boesten, J. 2010. Intersecting Inequalities: Women and Social Policy in Peru, 1990‐2000. University Park: Penn State University Press.

[fea212124-bib-0003] Cáceres, C. M. , M. Cueto , and N. Palomino . 2008. “Policies around Sexual and Reproductive Health and Rights in Peru: Conflict, Biases, and Silence.” Public Global Health 3 (S2): 39–57. 10.1080/17441690801981159 19288352

[fea212124-bib-0004] Cámara‐Reyes, R. , D. Obregón‐Gavilán , and M. Tipiani‐Mallma . 2018. “Aborto Terapeutico en el Peru: Una revision sobre el enfoque actual.” Revista Médica Panacea 7 (2): 74–7.

[fea212124-bib-0005] Castañeda, C. 2002. Figurations: Child, Bodies, Worlds. Durham, NC: Duke University Press:

[fea212124-bib-0006] Chaparro‐Buitrago, J. 2022. “Debilitated Lifewords: Women's Narratives of Forced Sterilization as Delinking from Reproductive Rights.” Medical Anthropology Quarterly 36 (2): 295–311. 35274360 10.1111/maq.12700 PMC9545704

[fea212124-bib-0007] de la Cadena, M. 2000. Indigenous Mestizos: The Politics of Race and Culture in Cuzco, Peru 1919‐1991. Durham, NC: Duke University Press.

[fea212124-bib-0008] Drucker, D. 2020. Contraception: A Concise History. Cambridge, MA: MIT Press.

[fea212124-bib-0009] Duffy, D. , C. Freeman , and S. Rodríguez . 2023. “Beyond the State: Abortion Care Activism in Peru.” Signs 48 (3): 609–34. 10.1086/723296 PMC7614643 37324651

[fea212124-bib-0010] Laverty, C. , and D. de Vos . 2021. “Reproductive Violence as a Category of Analysis: Disentangling the Relationship between ‘the Sexual’ and ‘the Reproductive’ in Transitional Justice.” International Journal of Transitional Justice 15 (3): 616–35.

[fea212124-bib-0011] Millar, E. 2017. Happy Abortions: Our Bodies in the Era of Choice. New York: Bloomsbury.

[fea212124-bib-0012] Murphy, M. 2017. The Economization of Life. Durham, NC: Duke University Press.

[fea212124-bib-0013] O'Shaughnessy, A. 2022. “Bodies of Change: Analyzing the Embodied and Affective Movement for Abortion Rights in Ireland.” PhD dissertation, University of Cambridge.

[fea212124-bib-0014] Roberts, D. 2015. “Reproductive Justice, Not Just Rights.” Dissent 62 (4): 79–82.

[fea212124-bib-0015] Ross, L. , and R. Solinger . 2017. Reproductive Justice: An Introduction. Oakland: University of California Press.

[fea212124-bib-0016] Seperak Viera, R. A. , L.P. Cerellino , J. E. Ochoa‐Luna , A. P. Torres‐valer Basauri , and C. M. Cáceres . 2019. “Maternidad en Perú a través del uso del Sentimient Analysis en Facebook.” Revista Latina de Comunicacion Social 74: 1031–55.

